# A cross‐linguistic test of automated speech and language analysis for detecting Alzheimer’s disease: Machine learning evidence from English and Spanish speakers

**DOI:** 10.1002/alz.084717

**Published:** 2025-01-09

**Authors:** Paula Andrea Perez‐Toro, Franco Javier Ferrante, Gonzalo Nicolás Pérez, Andrea Slachevsky, Elmar Nöth, Maria Schuster, Andreas Maier, Juan Rafael Orozco Arroyave, Adolfo M. Garcia

**Affiliations:** ^1^ Pattern Recognition Lab, Friedrich‐Alexander Universität, Erlangen‐Nürnberg, Erlangen Germany; ^2^ GITA Lab, Universidad de Antioquia, Medellín, Antioquia Colombia; ^3^ Massachusetts General Hospital and Harvard Medical School, Boston USA; ^4^ Cognitive Neuroscience Center, University of San Andrés, Victoria, Buenos Aires Argentina; ^5^ Neurology Department, Hospital del Salvador, University of Chile, Santiago Chile; ^6^ Ludwig‐Maximilians University, Munich Germany; ^7^ Universidad Santiago de Chile, Estación Central, Santiago de Chile Chile; ^8^ Global Brain Health Institute, University of California, San Francisco, CA USA

## Abstract

**Background:**

Digital health research on Alzheimer’s disease (AD) points to automated speech and language analysis (ASLA) as a globally scalable approach for diagnosis and monitoring. However, most studies target uninterpretable features in Anglophone samples, casting doubts on the approach’s clinical utility and cross‐linguistic validity. The present study was designed to tackle both issues.

**Method:**

The study included 178 native English speakers from the Pitt corpus (134 with AD, 44 controls) and 39 native Spanish speakers from a Chilean cohort (21 with AD, 18 controls). Participants were audio‐recorded as they described the Cookie Theft picture. Recordings were used to extract speech timing features (reflecting semantic memory retrieval effort), whereas their transcriptions were used to derive vocabulary selection features (revealing lexicon navigation patterns). We first trained classifiers with English‐speaking AD patients and controls, and then tested them in (a) a within‐language setting (with English‐speaking patients and controls as testing folds) and (b) a between‐language setting (with Spanish‐speaking patients and controls as testing folds). To discriminate between groups, in each case, we ran a separate classifier per modality (timing, vocabulary) and then another combining both modalities via early fusion. Finally, we explored whether the most sensitive features in each setting could predict patients’ clinical severity, as indexed through the Mini‐Mental Status Examination (MMSE).

**Result:**

Within‐language analysis showed that discrimination between AD patients and controls was good when based on speech timing (AUC = 0.75) and vocabulary (AUC = 0.79) features alone, and excellent when upon combining both modalities (AUC = 0.91). Between‐language analyses yielded maximal discrimination for the speech timing classifier (AUC = 0.79), surpassing vocabulary features (AUC = 0.60), and the fusion of both dimensions (AUC = 0.66). Patients’ MMSE scores were robustly predicted by speech timing features in both the within‐language (ρ = 0.41, p < .001) and the between‐language (ρ = 0.60, p < .001) settings.

**Conclusion:**

Interpretable ASLA features capturing semantic memory processes can robustly identify AD patients, with speech timing measures showing greater potential for cross‐linguistic generalization and severity prediction. Our approach paves the way for novel studies bridging the gap between ASLA and global approaches to dementia.

